# ChatGPT in the Management of Chronic Rhinosinusitis with Nasal Polyps: Promising Support or Digital Illusion? Insights from a Multicenter Observational Study

**DOI:** 10.3390/jcm14134501

**Published:** 2025-06-25

**Authors:** Riccardo Manzella, Angelo Immordino, Cosimo Galletti, Federica Giammona Indaco, Giovanna Stilo, Giuliano Messina, Francesco Lorusso, Rosalia Gargano, Silvia Frangipane, Giorgia Giunta, Diana Mariut, Daniele Portelli, Patrizia Zambito, Maria Grazia Ferrisi, Francesco Ciodaro, Manuela Centineo, Salvatore Maira, Francesco Dispenza, Salvatore Gallina, Ignazio La Mantia, Francesco Galletti, Bruno Galletti, Federico Sireci

**Affiliations:** 1 Otorhinolaryngology Unit, Biomedicine, Neuroscience and Advanced Diagnostic Department, University of Palermo, 90127 Palermo, Italy; riccardo.manzella@community.unipa.it (R.M.); francesco.lorusso@policlinico.pa.it (F.L.); rosalia.gargano@unipa.it (R.G.); francesco.dispenza@unipa.it (F.D.); salvatore.gallina@unipa.it (S.G.); 2 Faculty of Medicine and Surgery, University of Enna “Kore”, 94100 Enna, Italy; cosimo.galletti01@unikore.it; 3 Otorhinolaryngology Unit, Umberto I Hospital, 94100 Enna, Italy; federicagiammonaindaco@gmail.com (F.G.I.); patrizia.zambito@asp.enna.it (P.Z.); salvatore.maira@asp.enna.it (S.M.); 4 Otorhinolaryngology Unit, Department of Medical-Surgical Sciences and Advanced Technologies, University of Catania, 95131 Catania, Italy; giovastilo@gmail.com (G.S.); giuliano.messina1@studium.unict.it (G.M.); uni383422@studium.unict.it (S.F.); giorgia.giunta@studium.unict.it (G.G.); dianacatalina.mariut@studium.unict.it (D.M.); i.lamantia@unict.it (I.L.M.); 5 Otorhinolaryngology Unit, Department of Adult and Development Age Human Pathology, University of Messina, 98122 Messina, Italy; danieleportelli@yahoo.it (D.P.); mariagrazia.ferrisi@gmail.com (M.G.F.); francesco.ciodaro@polime.it (F.C.); francesco.galletti@unime.it (F.G.); bruno.galletti@polime.it (B.G.); 6 Digital Consultant Freelance, 90100 Palermo, Italy; m.centineo@hotmail.it; 7 Otorhinolaryngology Unit, Department of Precision Medicine in Medical, Surgical and Critical Care, University of Palermo, 90127 Palermo, Italy; federico.sireci@unipa.it

**Keywords:** artificial intelligence, ChatGPT, chronic rhinosinusitis, nasal polyps, biological therapy, monoclonal antibodies

## Abstract

**Background/Objective:** Chronic rhinosinusitis with nasal polyps is a chronic inflammatory disease with a significant impact on quality of life and is frequently associated, from a pathogenetic perspective, with type 2 inflammation. The introduction of biologic therapies has marked a turning point in the management of severe forms of the disease, offering a valuable treatment option. However, selecting the most suitable biologic agent for a specific patient remains a clinical challenge. Artificial intelligence, and, in particular, ChatGPT, has recently been proposed as a potential tool to support medical decision-making and guide therapeutic choices. To evaluate the concordance between the therapeutic recommendations provided by ChatGPT and those of a multidisciplinary expert board in selecting the most appropriate biologic therapy for CRSwNP patients, based on the analysis of their phenotype and endotype. **Methods:** A multicenter observational cohort study was conducted. Clinical data from 286 patients with CRSwNP were analyzed. For each case, the therapeutic choice among Dupilumab, Mepolizumab, and Omalizumab was compared between the board and ChatGPT. Concordance rates and Cohen’s Kappa coefficient were calculated. **Results:** Overall concordance was 59.2%, with a Cohen’s Kappa coefficient of 0.116. Concordance by drug was 62.8% for Dupilumab, 26.5% for Mepolizumab, and 9.1% for Omalizumab. Patients presented with severe clinical profiles, with an average Nasal Polyp Score of 6.22 and an average SNOT-22 score of 64.5. **Conclusions:** This study demonstrates that, despite its substantial theoretical potential, ChatGPT is currently not a reliable tool for the autonomous selection of biological therapies in patients with CRSwNP. Further studies are necessary to enhance its reliability and integration into clinical practice.

## 1. Introduction

In the evolving landscape of medical research and clinical care, the integration of artificial intelligence (AI) represents a transformative shift. This change is driven by the increasing availability of digital health data and the urgent need for more efficient, personalized, and evidence-based approaches to diagnosis and therapy.

In recent years, artificial intelligence (AI) has taken on an increasingly prominent role in the medical field, offering new perspectives for the diagnosis, treatment, and management of chronic diseases. Its applications span diverse areas, from image recognition and predictive analytics to virtual health assistants and decision support systems. This growing interest is due to its ability to process large datasets and generate responses using complex language models. Among the most innovative tools is ChatGPT, a generative language model developed by OpenAI, based on transformer architecture and trained on an extensive corpus of textual data. Now in its fourth version, the model can process natural language queries and generate structured, contextualized responses that align with current scientific evidence [1].

ChatGPT was initially conceived for theoretical and educational medical applications, but its applications have rapidly extended into clinical settings. It is capable of interpreting clinical scenarios, simulating medical reasoning, and even assisting in decision-making processes, making it a valuable aid for healthcare professionals in various contexts.

In healthcare, ChatGPT is employed to simplify complex medical information, support the education of students and residents, assist in drafting clinical documentation, and even simulate diagnostic and therapeutic scenarios [2].

Its potential utility has been demonstrated in several medical specialties. For instance, in cardiology, the model has shown promising results in interpreting and explaining electrocardiogram reports and managing chronic conditions. It has also been used in patient education programs to address risk factors and promote behavioral recommendations for cardiovascular disease prevention [3].

In neurology, ChatGPT has been tested for data management, decision-making support, and as educational guidance. It has also been useful in simplifying complex concepts such as diagnostic procedures and therapeutic strategies in neurodegenerative diseases [4].

In oncology, it has been proposed as a tool to provide patients with information about different types of cancer, prevention, risk factors, diagnosis, treatments, and side effects. It has also been considered for emotional and psychological support for patients and their families [5].

In pediatrics, ChatGPT has supported pediatricians in interpreting symptoms and assisting in clinical decision making. It is also useful for answering parental questions regarding the vaccination schedule and child development [6].

Dermatologists use AI to provide information about skincare routines, guide patients in recognizing skin problems, and help them understand when to consult a specialist [7].

Despite these promising developments, the integration of ChatGPT in specialty fields such as otolaryngology remains relatively unexplored. Only limited research has addressed how language models may support the management of complex inflammatory conditions affecting the upper airways.

However, in the field of otolaryngology, the application of ChatGPT is still in its early stages. In particular, chronic rhinosinusitis with nasal polyps (CRSwNPs) remains a complex therapeutic challenge requiring a personalized approach tailored to the predominant inflammatory endotype. CRSwNP involves persistent inflammation of the nasal and paranasal sinus mucosa, with symptoms lasting more than 12 weeks. The inflammation leads to a typical clinical presentation characterized by anterior rhinorrhea, post-nasal drip, reduced sense of smell and taste, nasal congestion, and facial pressure [8].

It is a common condition, with a global prevalence of around 5%, and a significant impact on quality of life. CRSwNP is typically associated with type 2 inflammation involving both innate and adaptive immunity. Specifically, there is an increased presence of IL-2 cells, Th2 cells, and cytokines such as IL-4, IL-5, and IL-13, resulting in systemic and tissue eosinophilia and elevated IgE levels [9]. This type of inflammation is commonly found in CRSwNP comorbidities such as bronchial asthma and atopy, which contribute to the complexity of the clinical picture.

In a smaller subset of cases, a Th1/Th17-mediated inflammation is present, characterized by cytokines such as IL-17A, IL-8, IFN-γ, and neutrophilic predominance.

In recent years, the availability of biologic therapies such as Dupilumab, Mepolizumab, and Omalizumab has marked a significant therapeutic advancement for patients with inadequate response to systemic corticosteroids or endoscopic surgery. However, the lack of definitive predictive biomarkers complicates the selection of the most appropriate drug, increasing the risk of ineffective or economically unsustainable treatments.

Therefore, an urgent unmet clinical need exists for tools that can assist physicians in making personalized therapeutic choices based on the specific inflammatory profile and clinical characteristics of each patient.

In this context, AI tools like ChatGPT could theoretically support the selection of biologic therapies, including Dupilumab, Mepolizumab, and Omalizumab [10].

Nonetheless, the reliability and accuracy of such tools in replicating specialist clinical decision making remain to be validated.

To the best of our knowledge, no previous study has systematically assessed the capability of ChatGPT to recommend biologic treatments for CRSwNP in real clinical scenarios. The present study addresses this gap by evaluating whether ChatGPT’s recommendations align with those made by a multidisciplinary expert board.

This study aims to explore the potential of ChatGPT (version 4.0) in selecting the most appropriate biologic treatment for patients with CRSwNP, comparing its recommendations with those of a multidisciplinary expert board, in light of the most recent scientific evidence.

## 2. Materials and Methods

### 2.1. Study Sample

A multicenter prospective observational cohort study was conducted in Sicily between June 2023 and October 2024, involving patients assessed by otolaryngologists specialized in rhinology and by resident physicians who formed the so-called Rhinology Board. The hospitals involved in data collection were: the Otorhinolaryngology Unit of the University Hospital “Policlinico Paolo Giaccone” in Palermo, the Otorhinolaryngology Unit of the University Hospital “G. Martino” in Messina, and the Otorhinolaryngology Unit of the University Hospital “G. Rodolico” in Catania.

The aim of the present study was to identify the most appropriate biologic treatment—selected collaboratively among the available options (Dupilumab, Mepolizumab, and Omalizumab)—and prescribed in accordance with the criteria approved by the “Agenzia Italiana del Farmaco” (AIFA) for the management of CRSwNP.

Anamnestic data were collected, including variables such as age, sex, associated comorbidities and concomitant clinical conditions (including atopic dermatitis and bronchial asthma), allergy history, nonsteroidal anti-inflammatory drug (NSAID) hypersensitivity, use of intranasal corticosteroids (INCSs), systemic corticosteroid (SCS) therapy, previous endoscopic sinus surgeries, number of prior surgical procedures, serum immunoglobulin-E levels (IgE, expressed in kU/L), circulating eosinophil count (cells/mm^3^), scores from the Sinonasal Outcome Test-22 (SNOT-22), Nasal Polyp Score (NPS), and results from the “Sniffin’ Sticks” olfactory test.

The inclusion criteria were those established by AIFA for access to biologic therapy in patients with CRSwNP in Italy: diagnosis of severe CRSwNP (defined by NPS ≥ 5 and/or SNOT-22 ≥ 50), documented failure of prior medical therapy with corticosteroids (due to ineffectiveness or adverse effects) and/or failure of endoscopic sinus surgery (due to complications or ineffectiveness), and no concurrent treatment with other biologic drugs.

The study sample included all patients aged 18 years or older with a diagnosis of CRSwNP.

Exclusion criteria included diagnosis of eosinophilic granulomatosis with polyangiitis (EGPA), poor adherence to pharmacological therapy, pregnancy, individuals under 18 years of age, previous treatment with radiotherapy or chemotherapy within the 12 months prior to enrollment, and patients undergoing chronic systemic corticosteroid therapy for concomitant autoimmune disease. Only patients who had completed a follow-up of at least 12 months were included in this study. All patients provided informed consent before participation. Clinical data were collected at baseline (prior to biologic treatment initiation) through a standardized case report form (CRF) shared across all centers, ensuring uniformity in data acquisition. The CRF included demographic information, comorbidities, clinical history, and biomarker values, as well as previous treatments and surgeries. Each patient underwent baseline assessment, including nasal endoscopy (for NPS evaluation), completion of the SNOT-22 questionnaire, and the Sniffin’ Sticks olfactory test.

Biologic therapy was initiated within four weeks of baseline assessment, and patients were monitored longitudinally with scheduled follow-up visits at 1, 3, 6, and 12 months. At each follow-up visit, the same set of evaluations was repeated (NPS, SNOT-22, and olfactory test), and any adverse events or changes in comorbid conditions were documented.

All data were entered into a centralized anonymized database hosted on a secure institutional server. Quality control checks were performed monthly by a data monitoring committee composed of representatives from each center. Missing data were addressed through direct consultation with the treating clinicians, and inconsistencies were resolved by consensus among the investigators. The study complied with the Declaration of Helsinki and was approved by the ethics committee.

### 2.2. Tools Used in Patient Evaluations

The SNOT-22 is a validated and widely used tool to assess the patient’s subjective perspective. This is a validated clinical questionnaire used to evaluate symptoms and quality of life in patients with chronic rhinosinusitis, with or without nasal polyps. It consists of 22 questions, each addressing various aspects of symptomatology such as nasal obstruction, discharge, facial pain, smell disturbances, sleep issues, and psychological impact. Specifically, items 1–12 assess physical symptoms, including rhinologic, ear-related, and facial symptoms, while items 13–22 evaluate quality of life by analyzing sleep function and psychological aspects.

Each question is scored from 0 (no symptoms) to 5 (very severe symptoms), for a total score ranging from 0 to 110. The higher the score, the greater the level of impairment perceived by the patient.

SNOT-22 is primarily used in otolaryngology to monitor the clinical progression of the disease and to assess the effectiveness of medical or surgical treatments. Moreover, it serves as a reference criterion for access to biologic therapies, representing an essential tool in both the objective and subjective evaluation of the disease’s impact on the patient’s daily life. Its widespread use is due to its simplicity, intuitiveness, and short completion time [11,12].

As a physician-assessed indicator, the NPS was used. NPS is a scoring system employed to quantify the presence and extent of nasal polyps in patients with CRSwNP. It is evaluated during a nasal endoscopic examination, and a score from 0 to 4 is assigned to each nasal cavity based on the size, origin, and obstruction caused by the polyps, yielding a total score ranging from 0 to 8 [13]. It is useful for assessing disease severity, response to pharmacological and/or surgical treatment, and determining eligibility for biologic therapy according to AIFA criteria.

Another important aspect to evaluate is hyposmia, which can be assessed using the Sniffin’ Sticks Test. This is a standardized psychophysical test used to assess olfactory function and, consequently, the degree of olfactory impairment—one of the most frequent and debilitating symptoms of the disease. The test uses pens containing odorants, and the patient must choose the correct answer from a multiple-choice format consisting of one correct option and three distractors. The test consists of 16 questions, and olfaction is considered normal with ≥12 correct responses. Patients scoring between 9 and 11 are considered hyposmic, while those scoring ≤8 are defined as anosmic. The test is used both in the initial diagnosis and for monitoring treatment response, allowing the quantification of improvements in olfactory function following therapy [10].

Regarding the interaction with ChatGPT, parameters were evaluated using ChatGPT (version 4.0). For each case, a separate chat session was used in which all collected clinical information was presented. ChatGPT was asked the following question: “What is the best biologic treatment among Dupilumab, Mepolizumab, and Omalizumab for this patient?” No identifiable patient information was shared with ChatGPT. The responses from ChatGPT were recorded. The same clinical information was provided to the Rhinology Board at each center, where the group of rhinology experts reviewed each case individually and reached a consensus on the most appropriate therapeutic strategy. The choice of the biologic therapy was made based on a multidimensional assessment that included: clinical profile and comorbidities (such as asthma, allergy, atopic dermatitis), availability and values of biomarkers (eosinophils, total IgE), response to previous treatments, number of administrations required for each drug (e.g., biweekly vs. monthly). Therapeutic decisions were made through internal clinical consensus at each center, without the need for unanimity. In the event of differing opinions, the approach supported by the majority of the senior clinicians was adopted. The treatment recommendations provided by the Rhinology Board were implemented in all cases and accepted by all patients. The concordance between the Rhinology Board’s recommendations and ChatGPT’s suggestions for biologic therapy was then evaluated.

### 2.3. Statistical Analysis

Statistical analysis was performed to assess the agreement between the therapeutic recommendations provided by ChatGPT (version 4.0) and those made by the multidisciplinary board in selecting the most appropriate biologic treatment for patients with CRSwNP. Continuous variables were described using mean and standard deviation, while categorical variables were expressed as absolute frequencies and percentages.

The concordance between the therapeutic choices proposed by ChatGPT and those of the Board was reported as the overall agreement percentage. To measure agreement beyond chance, Cohen’s Kappa coefficient was calculated—a statistical tool used to evaluate agreement between two raters on categorical classifications. Kappa values were interpreted according to the scale proposed by Landis and Koch: values < 0.20 indicate poor agreement, 0.21–0.40 fair agreement, 0.41–0.60 moderate agreement, 0.61–0.80 substantial agreement, and > 0.80 almost perfect agreement.

The analysis was conducted both overall and stratified according to drug type (Dupilumab, Mepolizumab, Omalizumab) to highlight potential differences in ChatGPT’s ability to identify the most appropriate treatment based on the patient’s clinical profile.

All data analyses were carried out using JASP statistical software (version 0.17.2, Amsterdam, The Netherlands), an open-source application designed to provide an intuitive interface and advanced tools for academic and clinical data analysis. The level of statistical significance was set at *p* < 0.05.

## 3. Results

A total of 286 patients were included in the study, of whom 173 were male (61.1%) and 110 female (38.9%). The mean age was 55.4 ± 13.5 years, with a range from 18 to 89 years.

To identify the presence of type 2 inflammation, serum IgE levels and blood eosinophil counts were evaluated. The mean serum IgE level was 248.2 ± 382.1 kU/L (range: 10–3656), while the mean blood eosinophil count was 578.3 ± 340.0 cells/mm^3^ (range: 10–2130).

The presence of common comorbidities sharing a type 2 inflammatory pattern was also assessed. A history of allergic disease was found in 171 patients (60.4%), atopic dermatitis in 57 patients (20.1%), hypersensitivity to nonsteroidal anti-inflammatory drugs in 70 patients (24.7%), and bronchial asthma in 182 patients (64.3%).

With regard to prior treatments, both surgical and medical therapies with topical and/or systemic corticosteroids were considered. Of all patients, 249 (88%) had been treated with intranasal corticosteroids, and 199 (70%) had received systemic corticosteroids. Endoscopic sinus surgery was performed in a total of 232 patients (82%). Specifically, 118 patients (41.7%) had undergone one surgical procedure, while 114 patients (40.3%) had undergone two or more procedures.

In terms of disease severity, the mean NPS was 6.22 ± 1.17, and the mean SNOT-22 score was 64.5 ± 18.4. Assessment using the Sniffin’ Sticks Test showed a mean score of 2.9 ± 3.5. Descriptive statistics of the study population are summarized in Table 1.

**Figure 1:**
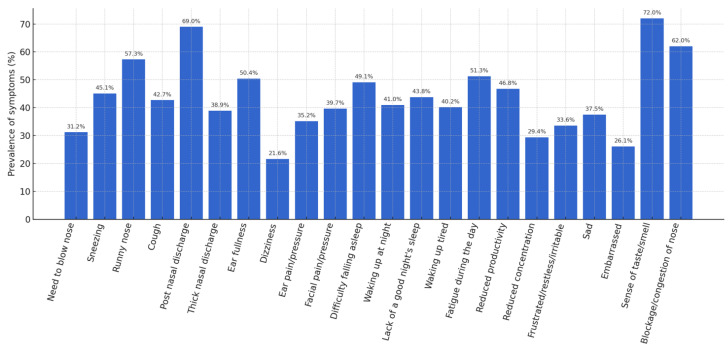
Prevalence of symptoms (%).

A detailed analysis of the individual items of the SNOT-22 made it possible to identify the most frequent symptoms. An altered sense of taste/smell was observed in 72% of patients, post-nasal drip in 69%, nasal obstruction in 62%, and anterior rhinorrhea in 57.3%. Figure 1 shows the prevalence of these symptoms.

**Figure 2:**
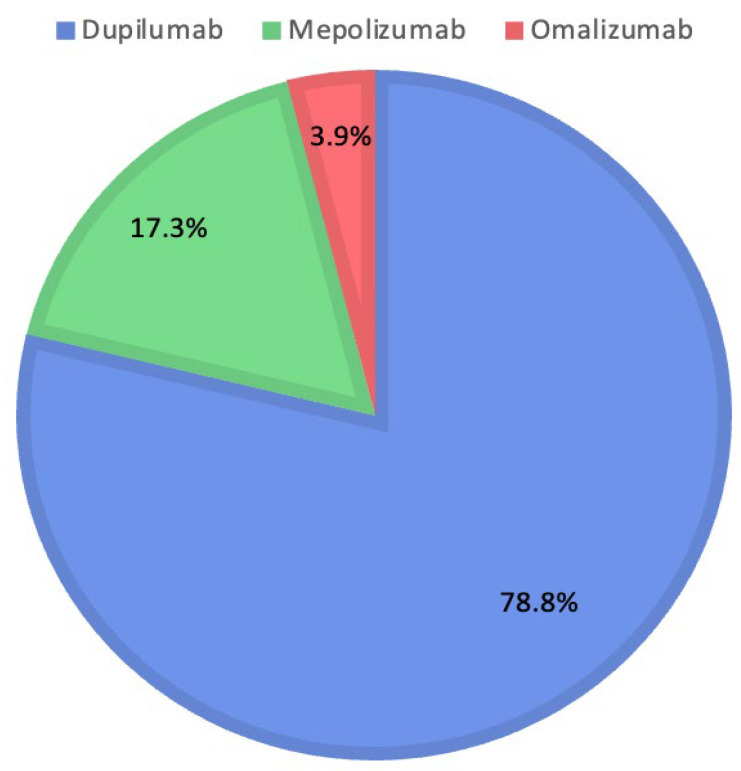
Distribution of medications prescribed by the Rhinology Board.

The therapeutic decisions of the Rhinology Board include the use of Dupilumab in 223 patients (78.8%), Mepolizumab in 49 patients (17.3%), and Omalizumab in 11 patients (3.9%). Figure 2 shows the percentages for each biologic drug selected by the Rhinology Board.

**Figure 3:**
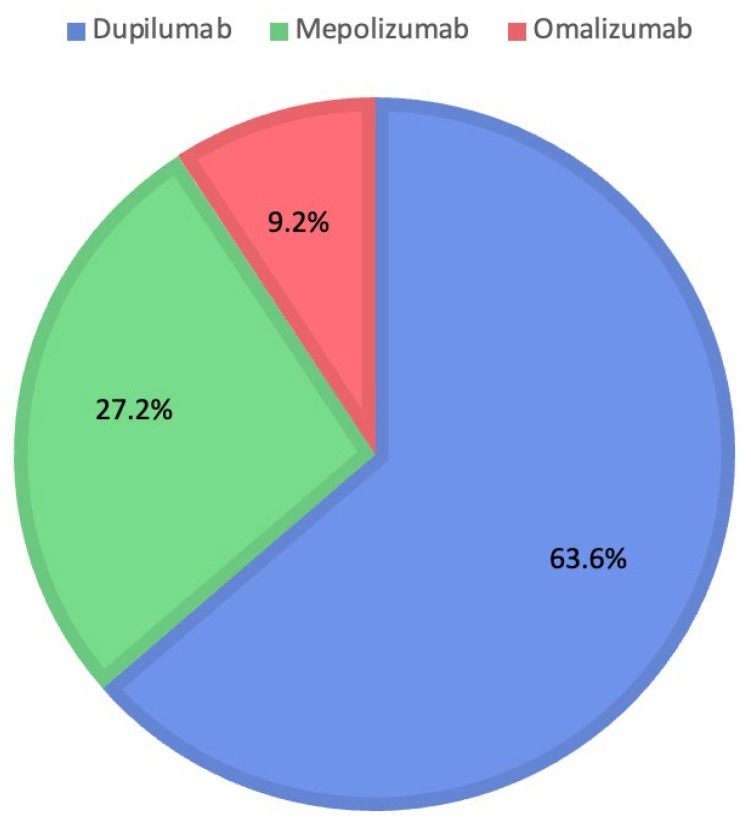
Distribution of medications prescribed by ChatGPT.

Instead, evaluating ChatGPT’s decisions shows the choice of Dupilumab for 180 patients (63.6%), Mepolizumab for 77 patients (27.2%), and Omalizumab for 26 patients (9.2%). Figure 3 shows the percentages for each biologic drug chosen by ChatGPT.

**Figure 4:**
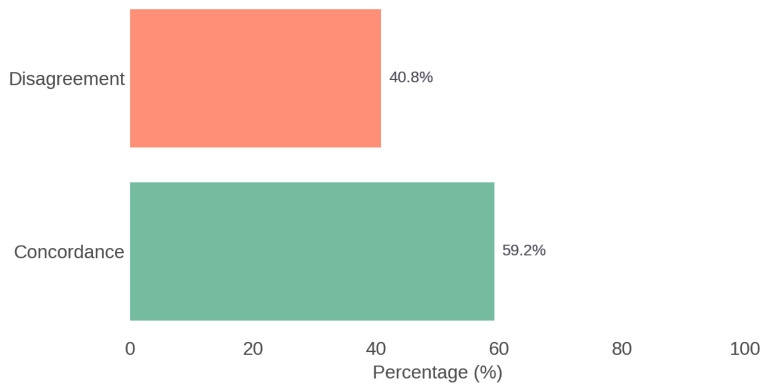
Overall concordance between the Rhinology Board and ChatGPT.

The therapeutic agreement between the recommendations of the multidisciplinary Board and those generated by ChatGPT was 59.2%. The analysis using Cohen’s Kappa coefficient showed a value of 0.116, indicating poor agreement. Figure 4 illustrates the overall concordance between the Rhinology Board and ChatGPT.

All treatment decisions were made in accordance with the Rhinology Board’s recommendations, and ChatGPT’s suggestions did not influence any follow-up decisions.

**Figure 5:**
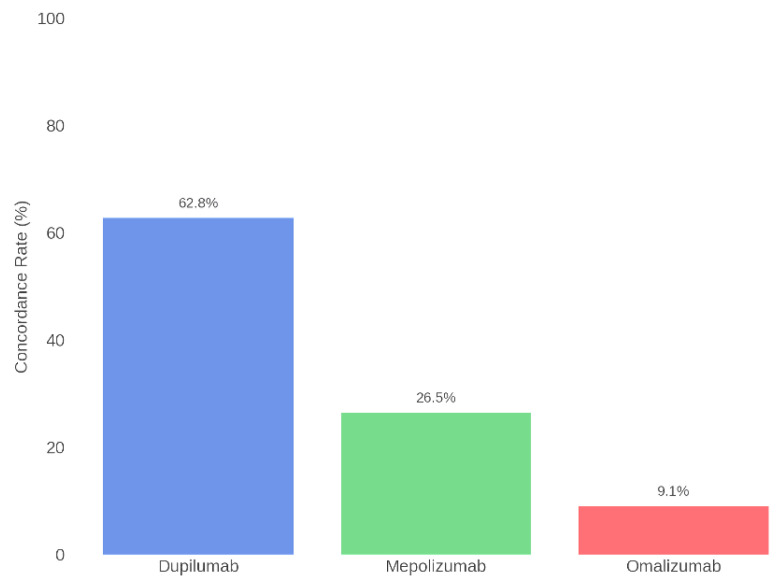
Therapeutic concordance per drug: Rhinology Board vs. ChatGPT.

Subsequently, concordance was also assessed for each individual biologic therapy. Specifically, the highest agreement was found for Dupilumab, with alignment between the Board and ChatGPT in 62.8% of cases. For Mepolizumab, concordance was 26.5%, while for Omalizumab it was 9.1%. Table 2 and Figure 5 present the concordance data for each specific therapy.

Clinical outcomes were also evaluated at 6 and 12 months to assess treatment effectiveness. Patients treated with Dupilumab showed the most pronounced improvements. The mean SNOT-22 score decreased from 64.5 (±18.4) at baseline to 33.0 (±12.5) at 6 months (a 49% reduction), and further improved to 28.5 (±11.7) at 12 months. The Nasal Polyp Score (NPS) similarly decreased from 6.22 (±1.17) at baseline to 3.10 (±1.05) at 6 months and 2.75 (±1.00) at 12 months. Olfactory function, measured by the Sniffin’ Sticks Test, improved markedly from 2.9 (±3.5) at baseline to 6.5 (±3.0) at 6 months and 7.2 (±3.2) at 12 months. Patients receiving Mepolizumab also showed significant improvements, although to a lesser extent. The SNOT-22 score decreased from 64.5 (±18.4) to 38.5 (±14.0) at 6 months (−40%) and to 34.0 (±13.5) at 12 months (−47%). The NPS decreased from 6.22 (±1.17) to 3.60 (±1.10) at 6 months and 3.25 (±1.08) at 12 months. Olfactory scores improved from 2.9 (±3.5) to 5.5 (±3.2) at 6 months and 6.0 (±3.1) at 12 months. Omalizumab treatment resulted in a 38% decrease in SNOT-22 scores, from 64.5 (±18.4) to 40.0 (±15.0) at 6 months and to 36.0 (±14.0) at 12 months (−44%). The NPS decreased from 6.22 (±1.17) to 3.80 (±1.20) at 6 months and 3.50 (±1.15) at 12 months. Olfactory test scores improved from 2.9 (±3.5) to 5.3 (±3.0) at 6 months and 5.8 (±3.2) at 12 months.

## 4. Discussion

Historically, chronic rhinosinusitis (CRS) has been classified into two main forms: with nasal polyps (CRSwNP) and without nasal polyps (CRSsNPs). The pathogenesis of CRSsNPs is generally associated with a type 1 immune response, while CRSwNP is typically characterized by type 2 inflammation. In Western populations, type 2 inflammation is present in approximately 80% of CRSwNP cases. However, an overlap between the two pathogenic mechanisms is not uncommon, making it essential during the diagnostic phase to perform detailed clinical and instrumental evaluations in order to accurately identify the patient’s phenotype first and then the endotype [14].

Confirmation of type 2 inflammation can be achieved by assessing systemic eosinophilia and serum IgE levels. According to the literature and clinical guidelines, relevant cut-offs are as follows: eosinophils > 250/mm^3^ and total IgE > 100 kU/L. The combination of phenotypic analysis (based on treatment response) with endotypic profiling (through markers such as eosinophils, cytokines, IgE, T-helper cells, periostin, etc.) currently represents the most effective method to predict disease progression and post-surgical prognosis [14].

With the advent of biological therapies, identifying the patient’s endotype has become central to therapeutic decision making. The biologic therapies currently approved in Italy for CRSwNP are as follows: Dupilumab, Omalizumab, and Mepolizumab [15]. Dupilumab was approved by AIFA in 2020 [16,17], Omalizumab in 2022 [18], and Mepolizumab in 2023 [19,20], for adult patients with severe CRSwNP (NPS ≥ 5 or SNOT-22 ≥ 50) that is not controlled by systemic corticosteroids (SCS) and/or surgery, and who are already on intranasal corticosteroids (INC).

Despite the increasing adoption of endotype assessment protocols, the selection of the most appropriate biologic remains complex and can lead to ineffective or economically unsustainable treatments [21,22]. In this context, the application of artificial intelligence (AI), and specifically of language models like ChatGPT, represents a promising frontier in supporting therapeutic decision making [2]. In our study, ChatGPT was used as a comparative tool for selecting biological therapy in patients with CRSwNP.

The analysis revealed an overall concordance between ChatGPT and the multidisciplinary Board of 59.2%, with a Cohen’s Kappa coefficient of 0.116, indicating poor agreement. This result is significantly lower than that reported by Sireci et al. (2024), who, in a smaller sample of 72 patients, observed a concordance rate of 68% with a Kappa coefficient of 0.69 [23]. The comparison between these two studies suggests that as the sample size and clinical complexity increase, ChatGPT’s ability to replicate the Board’s decisions tends to decline.

It is plausible that, in smaller and less complex cohorts, the algorithm aligns more easily with standardized clinical patterns. However, when case numbers and clinical heterogeneity grow, the limitations of AI become more evident, revealing a reduced ability to capture the clinical and contextual nuances that often inform medical decisions.

The highest concordance was observed for Dupilumab (62.8%), likely due to its broad range of indications, upstream mechanism of action, and extensive supporting literature. Conversely, concordance for Mepolizumab (26.5%) and Omalizumab (9.1%) was modest. This may reflect ChatGPT’s limited ability to identify more specific clinical profiles, such as marked eosinophilia or IgE-mediated allergies—conditions that require an integrated clinical assessment not reducible to simple quantitative parameters.

Patients treated with Dupilumab demonstrated the most substantial clinical improvements across all measured outcomes, including symptom severity, polyp burden, and olfactory function. Mepolizumab and Omalizumab were also associated with meaningful benefits, although the degree of improvement was comparatively less pronounced. These findings reinforce the therapeutic rationale adopted by the Rhinology Board, which favored Dupilumab in the majority of cases, and underscore the effectiveness of a specialist-driven approach in managing CRSwNP. Moreover, the outcome trends provide a clinical benchmark that can help contextualize the limited concordance observed with AI-based treatment suggestions such as those generated by ChatGPT. While current AI tools may not yet be suitable for independent therapeutic decision-making, they may have potential as adjunctive supports within expert-guided clinical workflows.

Looking ahead, integration between AI and diagnostic tools (e.g., imaging or molecular analysis) could enhance the precision of therapeutic recommendations. However, the clinical adoption of ChatGPT also raises ethical and medico-legal concerns related to prescribing responsibility, transparency in algorithmic decision making, and the protection of sensitive data. For now, AI should be regarded as a developing tool to be used cautiously and critically, with a view to a future in which it may truly support clinical judgment and contribute to personalized care.

## 5. Conclusions

The results of this multicentric study, involving a larger patient cohort compared to previous investigations, demonstrate that despite its high theoretical potential, ChatGPT is not yet a reliable tool to autonomously support the selection of biological therapy in patients with CRSwNP. The overall concordance rate between ChatGPT and the Rhinology Board was 59.2%, with a Cohen’s Kappa coefficient of 0.116, indicating poor agreement. These values are significantly lower than those reported in prior studies, such as Sireci et al. (2024), who observed a 68% concordance and a Kappa coefficient of 0.69 in a smaller sample (n = 72) [23]. This discrepancy suggests that as sample size and clinical complexity increase, ChatGPT’s ability to replicate human clinical decision making diminishes markedly. Although the algorithm has access to a vast corpus of medical knowledge, it appears unable to fully grasp the clinical and contextual nuances required for a truly personalized approach. Concordance was acceptable only for Dupilumab (62.8%) but dropped significantly for Mepolizumab (26.5%) and Omalizumab (9.1%), highlighting structural limitations in the model’s capacity to distinguish between clinically distinct inflammatory profiles. These findings support the hypothesis that ChatGPT tends to favor recommendations based on generic patterns rather than detailed individual data analysis. Nonetheless, several limitations must be considered. The Rhinology Board’s decisions were based on comprehensive clinical evaluations and patient histories, which were not entirely captured in the standardized data provided to ChatGPT, potentially limiting the model’s accuracy. Furthermore, the study’s multicenter design was restricted to Sicilian centers, which may affect the generalizability of the findings. The retrospective and single-session nature of the ChatGPT evaluation, without the possibility of interactive dialogue, may have constrained its ability to handle complex cases. Additionally, excluding patients without a minimum 12-month follow-up could have introduced selection bias by omitting more challenging cases. Therefore, ChatGPT’s role should currently be confined to that of a non-autonomous decision-support tool, always used under specialist supervision. Future multicentric studies with larger cohorts, integrating advanced biological variables and more sophisticated AI models, will be essential to validate and refine the role of artificial intelligence in otolaryngology.

## Figures and Tables

**Table 1:** Descriptive statistics of the population.

Population (n = 286)	
Age (years)	55.4 ± 13.5
Sex	M = 173 (61.1%) F = 110 (38.9%)
Allergy	171 (60.4%)
NSAIDs allergy	70 (24.7%)
Atopic Dermatitis	57 (20.1%)
Asthma	182 (64.3%)
INC	249 (88%)
SCS	199 (70%)
SNOT-22	64.5 ± 18.4
NPS	6.22 ± 1.17
Sniffin’ stick test	2.9 ± 3.5
Eosinophilia	578.3 ± 340.0 × mm^3^
IgE	248.2 ± 382.1 kU/L
Previous ESS	232 (82%)

M = male; F = female; NSAIDs = nonsteroidal anti-inflammatory drugs; INC = intranasal corticosteroids; SCS = systemic corticosteroids; SNOT-22 = Sinonasal Outcome Test-22; NPS = Nasal Polyp Score; ESS = endoscopic sinus surgery.

**Table 2:** Therapeutic concordance per drug: Rhinology Board vs. ChatGPT.

Drug	Concordance (%)	Agreed Patients	Total Patients
Dupilumab	62.8%	140	223
Mepolizumab	26.5%	13	49
Omalizumab	9.1%	1	11

## Data Availability

No new data were created or analyzed in this study. Data sharing is not applicable to this article.

## Abbreviations

The following abbreviations are used in this manuscript:

AI	Artificial Intelligence
CRSwNP	Chronic rhinosinusitis with nasal polyps
AIFA	Agenzia Italiana del Farmaco
NSAID	Non-steroidal anti-inflammatory drug
INCS	Intranasal corticosteroids
SCS	Systemic corticosteroid
SNOT-22	Sinonasal Outcome Test-22
NPS	Nasal Polyp Score
EGPA	Eosinophilic granulomatosis with polyangiitis
ESS	Endoscopic sinus surgery
